# The Role of Vasopressin V2 Receptor in Drug-Induced Hyponatremia

**DOI:** 10.3389/fphys.2021.797039

**Published:** 2021-12-10

**Authors:** Sua Kim, Chor Ho Jo, Gheun-Ho Kim

**Affiliations:** ^1^Institute of Biomedical Science, Hanyang University College of Medicine, Seoul, South Korea; ^2^Department of Internal Medicine, Hanyang University College of Medicine, Seoul, South Korea

**Keywords:** aquaporin-2, collecting duct, nephrogenic syndrome of inappropriate antidiuresis, syndrome of inappropriate ADH secretion, vasopressin, water

## Abstract

Hyponatremia is frequently encountered in clinical practice and usually induced by renal water retention. Many medications are considered to be among the various causes of hyponatremia, because they either stimulate the release of arginine vasopressin (AVP) or potentiate its action in the kidney. Antidepressants, anticonvulsants, antipsychotics, diuretics, and cytotoxic agents are the major causes of drug-induced hyponatremia. However, studies addressing the potential of these drugs to increase AVP release from the posterior pituitary gland or enhance urine concentration through intrarenal mechanisms are lacking. We previously showed that in the absence of AVP, sertraline, carbamazepine, haloperidol, and cyclophosphamide each increased vasopressin V2 receptor (V2R) mRNA and aquaporin-2 (AQP2) protein and mRNA expression in primary cultured inner medullary collecting duct cells. The upregulation of AQP2 was blocked by the V2R antagonist tolvaptan or protein kinase A (PKA) inhibitors. These findings led us to conclude that the nephrogenic syndrome of inappropriate antidiuresis (NSIAD) is the main mechanism of drug-induced hyponatremia. Previous studies have also shown that the V2R has a role in chlorpropamide-induced hyponatremia. Several other agents, including metformin and statins, have been found to induce antidiuresis and AQP2 upregulation through various V2R-independent pathways in animal experiments but are not associated with hyponatremia despite being frequently used clinically. In brief, drug-induced hyponatremia can be largely explained by AQP2 upregulation from V2R-cAMP-PKA signaling in the absence of AVP stimulation. This paper reviews the central and nephrogenic mechanisms of drug-induced hyponatremia and discusses the importance of the canonical pathway of AQP2 upregulation in drug-induced NSIAD.

## Pathogenesis of Hyponatremia and Renal Action of Vasopressin

**Figure 1:**
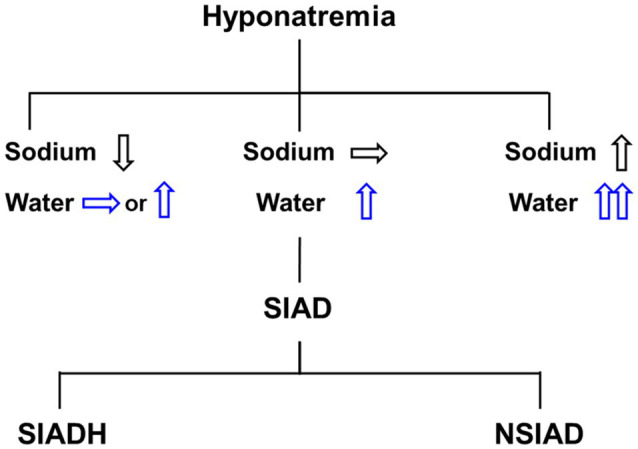
Differential diagnosis of hyponatremia. Hyponatremia may include water and sodium balance disorder, and pure water excess presents clinically with euvolemic hyponatremia. The syndrome of inappropriate antidiuresis (SIAD) is the most common cause of euvolemic hyponatremia and can be classified into the syndrome of inappropriate antidiuretic hormone secretion (SIADH) and the nephrogenic syndrome of inappropriate antidiuresis (NSIAD).

Hyponatremia, defined as a serum sodium concentration <135 mmol/L, is caused by an excess of water relative to sodium in the extracellular fluid. Although sodium depletion may precede water retention, primary water excess can occur irrespective of sodium balance (Figure 1). Water is retained in the body as a result of excessive intake and/or reduced renal excretion. The former is called primary polydipsia, usually occurring in neuropsychiatric patients. The latter can be induced by an absolute decrease in glomerular filtration rate (i.e., kidney failure) or abnormally increased water reabsorption along the renal tubule.

Renal water excretion is regulated by the action of arginine vasopressin (AVP), which is stored and released from the posterior pituitary gland. Synthesis of AVP in the hypothalamus is induced by both osmotic and non-osmotic stimuli, such as acute systemic hemodynamic changes, stress, and hypoxia (Schrier et al., 1979). In the kidney, the loop of Henle and collecting duct are the major sites of AVP action: AVP binds to the vasopressin V2 receptor (V2R) at the basolateral membrane of the collecting duct principal cells and induces osmotic water reabsorption through regulation of aquaporin-2 (AQP2) and aquaporin-3 (AQP3) water channel proteins (Jung and Kwon, 2016). The thick ascending limb of the loop of Henle plays an important role in countercurrent multiplication for urine concentration, and AVP strongly upregulates the expression of the Na-K-2Cl cotransporter 2 (NKCC2) of the thick ascending limb (Kim et al., 1999). The resultant outer medullary interstitial hypertonicity promotes osmotic water reabsorption along the collecting duct. On the other hand, inner medullary interstitial hypertonicity can be established by urea accumulation facilitated by urea transporters (UTs). By stimulating UT-A1 and UT-A3 in the inner medullary collecting duct and UT-A2 in the thin descending limb of Henle’s loop (Wade et al., 2000), AVP promotes urea recycling.

## The Syndrome of Inappropriate Antidiuresis As a Major Cause of Hyponatremia

Hyponatremia is the most common electrolyte disorder in hospitalized patients and is potentially life-threatening because of the risk of cerebral edema (Adrogué and Madias, 2000). The “syndrome of inappropriate secretion of antidiuretic hormone (SIADH)” is the most frequent cause of hyponatremia, and a variety of drugs can stimulate the release of AVP or potentiate its action (Ellison and Berl, 2007). Thus, most cases of drug-induced hyponatremia are considered to be consistent with a diagnosis of the SIADH when hyponatremia is associated with the use of a particular agent in hypo-osmolar and euvolemic conditions (Smith et al., 2000; Janicic and Verbalis, 2003). Theoretically, the SIADH can be diagnosed when AVP release is inappropriately elevated despite low plasma osmolality. However, the diagnosis of the SIADH is usually made after identifying several clinical and laboratory features without measurement of AVP (Ellison and Berl, 2007). Measurement of AVP concentrations may not be feasible in clinical practice and the results may be unreliable, because AVP is highly unstable in isolated plasma and the pre-analytic procedures are complicated (Bolignano et al., 2014).

Alternately, Dr. Robertson proposed a diagnosis of the “syndrome of inappropriate antidiuresis (SIAD),” given plasma AVP is actually suppressed in a certain proportion of patients diagnosed as SIADH (Robertson, 1989). The term “nephrogenic syndrome of inappropriate antidiuresis (NSIAD)” was also coined by Feldman et al. when they described two infants whose clinical and laboratory evaluations were consistent with the presence of the SIADH but had undetectable AVP because of gain-of-function mutations in the V2R (Feldman et al., 2005). Thus, the SIAD caused by renal water retention can be grouped into SIADH (with an excess of antidiuretic hormone) and NSIAD (with appropriately suppressed AVP secretion) according to its pathogenesis (Figure 1).

## Drug-Induced Hyponatremia: the Siadh Versus Nsiad

Table 1 presents the list of drugs that often cause hyponatremia in clinical practice. We classified them into AVP analogs, drugs that stimulate release of AVP, drugs that stimulate V2R in the kidney and induce the NSIAD, and others.

**Table 1:** Drugs that can cause hyponatremia.

AVP analogs
Desmopressin (dDAVP)
Oxytocin
Drugs that stimulate release of arginine vasopressin
Vincristine
Ifosfamide
Drugs that stimulate the vasopressin V2 receptor in the kidney
Chlorpropamide
Antidepressants: selective serotonin-reuptake inhibitors
Anticonvulsants: carbamazepine
Antipsychotics: haloperidol
Cyclophosphamide
Diuretics
Thiazides
Prostaglandin synthesis inhibitors
Nonsteroidal anti-inflammatory drugs
Cyclooxygenase-2 inhibitors

**Table 2:** Experimental antidiuretic agents without causing hyponatremia.

Phosphodiesterase-5 inhibition
Sildenafil
AMP-activated protein kinase (AMPK) activation
Metformin
β-Hydroxy β-methylglutaryl-CoA (HMG-CoA) reductase inhibition
Simvastatin
Lovastatin
Rosuvastatin
Cerivastatin
Fluvastatin
P2Y12 receptor antagonism
Clopidogrel
Epidermal growth factor receptor antagonism
Erlotinib
Azole antifungal agents
Fluconazole

## Avp Analogs

Desmopressin, a synthetic analog of AVP, has been prescribed for the treatment of diabetes insipidus and nocturnal polyuria. Although it is generally well tolerated, it can cause severe hyponatremia in susceptible patients because of its water-retaining effect (Kelleher and Henderson, 2006). Compared with AVP, desmopressin has a longer half-life and a greater antidiuretic effect caused by selective binding to the V2R (Kwon et al., 2013), but does not have unwanted vasopressor and uterotonic effects (Vilhardt, 1990). Together, V2R-mediated stimulation of adenylyl cyclases, elevation of cAMP, and activation of protein kinase A (PKA) are the canonical signaling pathways that trigger both increased AQP2 trafficking and AQP2 protein abundance (Jung and Kwon, 2016).

A meta-analysis indicated the incidence of desmopressin-induced hyponatremia was 7.6% in adults with nocturia (Weatherall, 2004). The development of hyponatremia during desmopressin use is likely dependent on the dose of desmopressin. However, we found that even low doses of desmopressin could induce hyponatremia in predisposed patients and that advanced age and lower hemoglobin were the risk factors for hyponatremia in adults using desmopressin for nocturnal polyuria (Choi et al., 2015).

Oxytocin, used to induce labor or abortion, may be associated with hyponatremia because of its antidiuretic activity. As expected, the risk of hyponatremia is increased when oxytocin is diluted in intravenous hypotonic fluids (Ahmad et al., 1975). Oxytocin and AVP are closely related peptides secreted from the posterior pituitary, and both are nine-amino-acid peptide hormones, of which seven are identical (Baribeau and Anagnostou, 2015). Oxytocin was shown to act as an antidiuretic hormone because oxytocin increases osmotic water permeability in perfused inner medullary collecting ducts isolated from Sprague–Dawley rats (Chou et al., 1995b), and its hydrosmotic action was mediated by the V2R (Chou et al., 1995a). In Sprague–Dawley rats, oxytocin treatment induced apical and basolateral translocation of AQP2 protein along the collecting duct. This response was blocked by pretreatment with a V2R antagonist (Jeon et al., 2003). The antidiuretic action of oxytocin was also demonstrated in humans in association with AQP2 upregulation (Joo et al., 2004). Taken together, pharmacological doses of oxytocin can induce antidiuretic effects as a result of V2R stimulation and subsequent AQP2 upregulation (Cheng et al., 2009).

### Drugs That Stimulate AVP Release

The anticancer chemotherapeutic agents, vincristine, vinblastine, cisplatin, and cyclophosphamide, were typically assumed to have stimulated release of AVP from the pituitary gland or to have increased production of AVP at the hypothalamus. However, evidence supporting these mechanisms is lacking (Berghmans, 1996).

Suskind et al. reported the case of a 3-year-old girl who was inadvertently administered an overdose of vincristine and developed clinical features compatible with the SIADH. Her blood vasopressin concentration was more than four times the normal value (Suskind et al., 1972). Stuart et al. showed that urinary vasopressin excretion was markedly elevated following administration of vincristine to a child with acute lymphatic leukemia (Stuart et al., 1975). Thus, the SIADH seems to be at the basis of vincristine-associated hyponatremia. Animal studies suggested that the SIADH may result from a direct toxic effect of vincristine on the neurohypophysis and the hypothalamic system (Uy et al., 1967; Robertson et al., 1973). In cases with increased plasma AVP concentration, however, dehydration due to vincristine toxicity or diarrhea needs to be differentiated from the SIADH (Jójárt et al., 1987).

Cyclophosphamide and ifosfamide are the representative alkylating agents that may be associated with hyponatremia. It is unclear whether plasma AVP concentrations are elevated following cyclophosphamide administration (Bressler and Huston, 1985; McCarron et al., 1995). On the other hand, elevated plasma AVP concentrations were found in a few cases of ifosfamide-induced hyponatremia (Cantwell et al., 1990; Kirch et al., 1997). Glezerman reported that ifosfamide-induced hyponatremia was corrected by the V2R antagonist conivaptan (Glezerman, 2009). This finding may support the possibility that the SIADH underlies ifosfamide-induced hyponatremia (Gross et al., 2011).

### Drugs That Stimulate V2R in the Kidney

Renal water retention may result from the direct effects of some medications on the collecting duct epithelium in the absence of an AVP-mediated mechanism of action. Traditionally, such medications were classified as drugs that potentiate the renal action of AVP; however, they are now thought to act specifically as V2R agonists to induce the NSIAD.

#### Chlorpropamide

Chlorpropamide is a long-acting first-generation sulfonylurea, previously used to treat diabetes mellitus type 2. Chlorpropamide also has antidiuretic effects and has been used to treat diabetes insipidus (Kunstadter et al., 1969); although not currently used in clinical practice, the antidiuretic mechanisms of chlorpropamide are worthy of review.

High doses of chlorpropamide are associated with hyponatremia, and this effect was described as the SIADH since the first report in 1970 (Fine and Shedrovilzky, 1970). However, administration of chlorpropamide did not augment release of AVP in humans or rats (Pokracki et al., 1981). While the SIADH was seen in 4% of patients receiving chlorpropamide in a clinic population, elevated plasma AVP concentrations were not demonstrated (Weissman et al., 1971).

Many studies have used the isolated toad urinary bladder to measure osmotic water permeability in response to chlorpropamide administration. Low concentrations of chlorpropamide enhanced the effect of AVP (Mendoza, 1969; Wales, 1971; Lozada et al., 1972; Hirji and Mucklow, 1991), whereas high concentrations increased water absorption across the membrane in the absence of AVP (Danisi et al., 1970; Urakabe et al., 1970; Ozer and Sharp, 1973; Mendoza and Brown, 1974).

Moses et al. investigated the mechanism by which chlorpropamide potentiates the action of AVP (Miller and Moses, 1970; Moses et al., 1982). They found that chlorpropamide enhanced the activity of renal medullary adenylate cyclase and increased renal medullary content of cAMP in response to desmopressin, supporting the concept that *in vivo* chlorpropamide acts at the V2R in the collecting duct to augment responsiveness to AVP (Moses et al., 1982). Other investigators have provided supporting evidence that chlorpropamide acts as a V2R agonist to exert its antidiuretic action. Chlorpropamide and AVP were first postulated to share a common site of action within the V2R in 1969 (Ingelfinger and Hays, 1969). Muta et al. (1989) showed that 1 mM chlorpropamide reduced AVP binding to the V2R within the rat renal tubular basolateral membrane in a competitive manner, indicating that chlorpropamide acts on the V2R (Muta et al., 1989). Using a radioiodinated derivative of AVP with high specific activity and high affinity for the V2R, Hensen et al. (1995) showed that low-dose oral chlorpropamide increased the V2R density without altering plasma AVP concentrations (Hensen et al., 1995); the V2R upregulation was therefore postulated to underlie chlorpropamide-induced hyponatremia.

#### Selective Serotonin Reuptake Inhibitors

A variety of antidepressants have been reported to be associated with hyponatremia: tricyclic antidepressants (TCAs), monoamine oxidase inhibitors, selective serotonin reuptake inhibitors (SSRIs), serotonin-norepinephrine reuptake inhibitors (SNRIs), and mirtazapine. These reports are supported by data from clinical and pre-clinical studies that indicate AVP plays an important role in the pathophysiology of major depression (Scott and Dinan, 2002). According to a cross-sectional study of elderly patients treated with antidepressants in the Netherlands, the prevalence of hyponatremia was 11.5% for the patients on TCAs, 10.2% for SSRI users, 8.6% for venlafaxine users, and 5.6% for patients using mirtazapine (Mannesse et al., 2013). Because non-suppressed plasma AVP levels were found in only a minority of these patients, the NSIAD was suggested as the underlying mechanism of SSRI-induced hyponatremia in most patients.

The mechanism of direct water retention from the kidney induced by SSRIs has been partly elucidated. Fluoxetine and sertraline are representative SSRIs that often associated with hyponatremia, causing significant morbidity and mortality (De Picker et al., 2014). An *in vitro* microperfused tubule study showed that in the absence of AVP, fluoxetine increased osmotic water permeability in the rat inner medullary collecting duct (IMCD). Furthermore, fluoxetine administration to rats for 10 days did not alter plasma AVP concentrations but increased AQP2 protein abundance in the kidney (Moyses et al., 2008).

We recently showed that in the rat IMCD, in the absence of vasopressin stimulation, sertraline upregulated AQP2 by inducing V2R-cAMP-PKA signaling (Kim et al., 2021). In IMCD suspensions, cAMP production was increased by sertraline and was attenuated by co-incubation with tolvaptan. In primary IMCD cell cultures, sertraline treatment increased total AQP2 and decreased phosphorylated AQP2 at S261. Notably, these responses were attenuated by co-incubation with tolvaptan or a PKA inhibitor. In addition, AQP2 membrane trafficking was induced by sertraline and blocked by co-incubation with tolvaptan or a PKA inhibitor. Furthermore, V2R and AQP2 mRNA expression and CREB-1 phosphorylation at S133 were induced by sertraline and blocked by co-incubation with tolvaptan. We concluded that sertraline acts as a V2R agonist in the kidney and leads to AQP upregulation by inducing AQP2 transcription and AQP2 dephosphorylation at S261 (Kim et al., 2021). Sertraline was reported to effectively reduce the number of wet episodes in adolescents with primary monosymptomatic enuresis who had experienced failure to desmopressin therapy (Mahdavi-Zafarghandi and Seyedi, 2014).

#### Carbamazepine

Carbamazepine and oxcarbazepine are the anticonvulsants most commonly reported to be associated with hyponatremia in epilepsy patients, although other anticonvulsants, such as eslicarbazepine, sodium valproate, lamotrigine, levetiracetam, and gabapentin, have also been reported to cause hyponatremia (Lu and Wang, 2017). The mechanism of anticonvulsant-associate hyponatremia has generally been considered to be inappropriate hypersecretion of AVP (Ashton et al., 1977; Smith et al., 1977), but an experimental study has indicated a direct effect of carbamazepine on the kidney through V2R stimulation without evidence of increased release of endogenous AVP (Meinders et al., 1975). Sekiya et al. also reported that 18-year-old male with carbamazepine-associated hyponatremia had features of the SIADH but had an undetectable level of plasma AVP and an elevated urine cyclic AMP excretion (Sekiya and Awazu, 2018). Thus, a human case of carbamazepine-induced NSIAD was demonstrated.

It has become clear that carbamazepine has a direct action on the collecting duct V2R, leading to AQP2 upregulation. *In vitro* microperfused tubule studies showed that in the absence of AVP, carbamazepine increased osmotic water absorption and AQP2 protein abundance in the rat IMCD by inducing the V2R-PKA pathway (de Bragança et al., 2010). We investigated the intracellular mechanisms of carbamazepine-induced AQP2 upregulation in the IMCD (Kim et al., 2021). In IMCD suspensions, cAMP production was increased by carbamazepine and was attenuated by co-incubation with tolvaptan. In primary IMCD cell cultures, incubation with carbamazepine increased the total AQP2 and decreased the phosphorylation of AQP2 at S261. Notably, these responses were reversed by co-incubation with tolvaptan or a PKA inhibitor. In addition, AQP2 membrane trafficking was induced by carbamazepine and blocked by co-incubation with tolvaptan or a PKA inhibitor. Furthermore, V2R and AQP2 mRNA expression and CREB-1 phosphorylation at S133 were induced by carbamazepine and blocked by co-incubation with tolvaptan. We concluded that carbamazepine acts as a V2R agonist in the kidney and leads to AQP upregulation by inducing AQP2 transcription and AQP2 dephosphorylation at S261 (Kim et al., 2021). Compatible with our results, carbamazepine was shown to have antidiuretic activity in seven out of nine patients with central diabetes insipidus (Wales, 1975).

Oxcarbazepine is a keto-analog of carbamazepine and may be also associated with hyponatremia. Sachdeo et al. investigated the mechanisms by which oxcarbazepine can lead to hyponatremia in epilepsy and healthy subjects (Sachdeo et al., 2002). They found that, after the water load, solute-free water clearance was diminished in both groups without a concomitant increase in the blood AVP concentrations. Thus, oxcarbazepine-induced hyponatremia was not attributable to the SIADH. It seems that oxcarbazepine and carbamazepine share the common mechanisms of the NSIAD, namely direct action on the V2R.

#### Haloperidol

Antipsychotic drugs can be grouped into first-generation antipsychotics (e.g., chlorpromazine, chlorprotixene, dixyrazine, flupentixol, fluphenazine, haloperidol, levomepromazine, melperone, perphenazine, prochlorperazine, thioridazine, or zuclopenthixole) and second-generation antipsychotics (e.g., aripiprazole, clozapine, olanzapine, paliperidone, quetiapine, risperidone, or ziprasidone). A Swedish population-based case–control study found an association between antipsychotic therapy and hospitalization due to hyponatremia. The association was stronger for first-generation antipsychotics than second-generation antipsychotics (Falhammar et al., 2019). Several psychotropic drugs have been reported to be associated with the features of the SIADH, but without demonstration of unsuppressed plasma AVP (Peck and Shenkman, 1979; Whitten and Ruehter, 1997; Bachu et al., 2006).

On the other hand, plasma AVP concentrations did not change significantly when haloperidol was given to seven normal volunteers at a dose level (1.0 mg i.m.) known to have central nervous system effects (Kendler et al., 1978). Thus, we assessed whether haloperidol can induce renal water retention in the absence of AVP stimulation. In IMCD suspensions, cAMP production was increased by haloperidol and was attenuated by co-incubation with tolvaptan. In primary IMCD cell cultures, haloperidol increased the total AQP2 and decreased the AQP2 phosphorylation at S261. Notably, these responses were attenuated by co-incubation with tolvaptan or a PKA inhibitor. In addition, AQP2 membrane trafficking was induced by haloperidol and blocked by co-incubation with tolvaptan or a PKA inhibitor. Furthermore, V2R and AQP2 mRNA expression and CREB-1 phosphorylation at S133 were induced by haloperidol and were blocked by co-incubation with tolvaptan. We concluded that haloperidol acts as a V2R agonist in the kidney and leads to AQP2 upregulation by inducing AQP2 transcription and AQP2 dephosphorylation at S261 (Kim et al., 2021).

#### Cyclophosphamide

Hyponatremia can be induced by various doses of cyclophosphamide during the treatment of malignancy and rheumatological disease (Lee et al., 2010). As described above, plasma AVP concentrations are not elevated in patients following the administration of intravenous cyclophosphamide (Bode et al., 1980; Bressler and Huston, 1985; Larose et al., 1987). Furthermore, antidiuresis was reported to occur in response to intravenous cyclophosphamide in patients with central diabetes insipidus (Campbell et al., 2000; Steinman et al., 2015), excluding the possibility of the SIADH.

We showed that in the rat IMCD, the active metabolite of cyclophosphamide (4-hydroperoxycyclophosphamide) increased cAMP production, AQP2 protein and mRNA expression, and V2R mRNA expression in the absence of vasopressin stimulation (Kim et al., 2015). These changes were significantly ameliorated by co-administration of tolvaptan, suggestive of V2R-mediated NSIAD.

### Thiazide Diuretics

Thiazide and loop diuretics are frequently used to treat edematous disorders. Although both classes of diuretic induce natriuresis, their effects on water balance may differ. Thiazides inhibit the Na-Cl cotransporter (NCC) in the distal convoluted tubule, the cortical diluting segment of the nephron. Thus, impairment of urine dilution and renal retention of water may be induced by thiazides (Hix et al., 2011). In contrast, loop diuretics, such as furosemide and torsemide, can inhibit the Na-K-2Cl cotransporter 2 (NKCC2) in the thick ascending limb, the outer medullary concentrating segment of the nephron. Thus, free-water clearance increases when urinary concentration is impaired by loop diuretics. Consequently, patients are prone to hyponatremia when using thiazides and hypernatremia when using loop diuretics.

Patients with thiazide-induced hyponatremia show features of the SIADH including low serum uric acid concentrations and increased fractional excretion of uric acid (Liamis et al., 2007). However, plasma AVP measurement in patients with thiazide-induced hyponatremia has produced conflicting results, with some studies reporting elevated AVP concentrations (Fichman et al., 1971; Luboshitzky et al., 1978), while others did not (Friedman et al., 1989; Frenkel et al., 2015; Ware et al., 2017). Ashraf et al. (1981) reported that plasma AVP was undetectable in metolazone-induced hyponatremia (Ashraf et al., 1981), suggestive of the NSIAD.

Thiazide-induced renal water retention may be independent of NCC inhibition in the distal convoluted tubule. No hyponatremia is found in Gitelman syndrome or Gitelman-mimic animals carrying a loss-of-function mutation in the NCC regulator Ste20 Proline-Alanine-rich Kinase (SPAK; Nadal et al., 2018). Hydrochlorothiazide administration resulted in reduced urine volume in lithium-treated NCC-knockout mice (Sinke et al., 2014). In particular, thiazides may act directly on the collecting duct, where water permeability is increased by vasopressin-independent mechanisms. Cesar and Magaldi performed *in vitro* microperfusion of IMCDs from AVP-deficient Brattleboro rats and showed that addition of hydrochlorothiazide to the perfusate enhanced osmotic water permeability (César and Magaldi, 1999). This effect was attenuated by adding prostaglandin E2 to the perfusate, suggestive of the involvement of prostaglandin signaling.

We investigated the antidiuretic effect of hydrochlorothiazide in rats with lithium-induced nephrogenic diabetes insipidus (NDI) and explored the regulatory responses of AQP2 in the collecting duct (Kim et al., 2004). In association with antidiuresis, hydrochlorothiazide treatment caused a significant partial recovery of AQP2 abundance after lithium-induced downregulation. We believe thiazide diuretics have a direct action on the collecting duct principal cells and induce AQP2 upregulation by modulating prostaglandin E2 signaling pathways. This postulated mechanism was recently supported by the findings of a genetic and phenotyping analysis, suggestive of a role for genetically determined prostaglandin-E2-mediated increased water permeability of the collecting ducts in the development of thiazide-induced hyponatremia (Ware et al., 2017). A subgroup of patients with thiazide-induced hyponatremia may carry a variant allele of the prostaglandin transporter SLCO2A1 gene that leads to reduced ability to transport prostaglandin E2 across the apical cell membrane; this reduction of prostaglandin E2 transport leads to increased luminal prostaglandin E2 and activates luminal EP4 receptors, causing membrane trafficking of AQP2 in the absence of AVP, directly reducing urine dilution and free-water excretion (Filippone et al., 2020).

### Prostaglandin Synthesis Inhibitors

Nonsteroidal anti-inflammatory drugs (NSAIDs), such as piroxicam, diclofenac, and indomethacin, are commonly used for pain control in daily clinical practice. They are rarely associated with hyponatremia, rather hyperkalemia and sodium retention with associated edema are much more frequently induced by NSAIDs (Raymond and Lifschitz, 1986); however, a few cases of severe NSAID-associated hyponatremia have been reported (Petersson et al., 1987). Prostaglandin E2 plays a critical physiologic and pathophysiologic role in inhibiting vasopressin action in the collecting duct (Breyer et al., 1990). Given NSAIDs inhibit prostaglandin synthesis, NSAIDs were thought to induce the SIADH (Demir et al., 2012); currently, potentiation of AVP action but not enhanced AVP release is considered as the most plausible explanation for NSAID-induced hyponatremia.

Prostaglandin E2 acts on the kidney through four different G-protein-coupled receptors, EP1-4 (Breyer and Breyer, 2001). In the presence of AVP, it can antagonize the renal AVP action *via* multiple EP receptors and signaling pathways (Breyer et al., 1990). However, prostaglandin E2 alone may increase collecting duct water permeability (Hébert et al., 1993). Olesen et al. showed that in the cortical collecting duct principal cells, EP2 and EP4 stimulation increased AQP2 apical membrane targeting, S256 phosphorylation, and S264 phosphorylation in the absence of AVP (Olesen et al., 2011). In addition, EP4 increases total kidney AQP2 protein abundance through an unknown mechanism (Li et al., 2009).

On the other hand, EP3 has an important role in the diuretic effects of prostaglandin E2. EP3 inhibits NKCC2 through coupling to Gi and reduces countercurrent multiplication in the medullary thick ascending limb. In the collecting duct principal cells, EP3 decreases AQP2 apical membrane targeting as a result of cAMP suppression or RhoA stimulation (Olesen and Fenton, 2013).

NSAID-mediated cyclooxygenase (COX) inhibition results in the blockade of the EP3 action, which contributes to their antidiuretic action (Breyer and Breyer, 2001). We previously showed that in lithium-induced NDI rats, treatment with COX-2 inhibitors reduced polyuria by upregulating AQP2 and NKCC2 expression in the collecting duct and thick ascending limb, respectively (Kim et al., 2008). The antidiuretic effect of NSAIDs or COX-2 inhibitors has been useful in the treatment of human NDI. Indomethacin was effective in reducing polyuria in lithium-induced NDI (Allen et al., 1989); however, COX-2 inhibitors including rofecoxib and celecoxib may be preferable, because of their superior antidiuretic action without induction of upper gastrointestinal side effects (Soylu et al., 2005).

## Experimental Antidiuretic Agents Without Causing Hyponatremia

**Figure 2:**
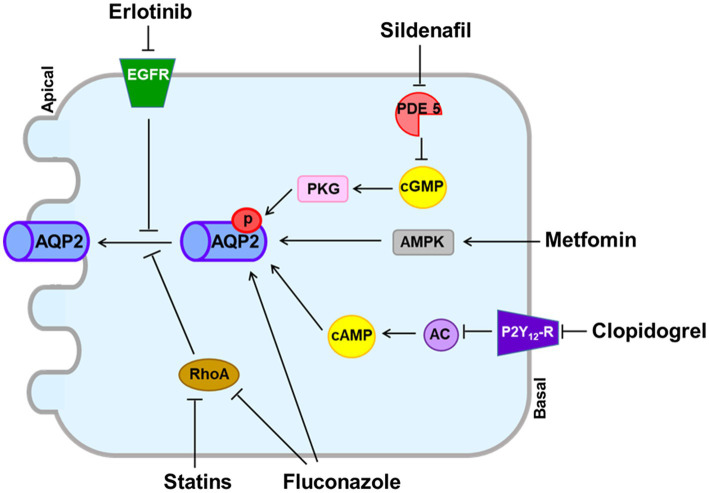
Vasopressin V2 receptor (V2R)-independent pathways for aquaporin-2 (AQP2) upregulation induced by experimental antidiuretic agents. The phosphodiesterase-5 inhibitor sildenafil citrate prevents degradation of cGMP, resulting in increased AQP2 expression in the apical membrane. Erlotinib inhibits the tyrosine kinase activity of epidermal growth factor receptor (EGFR) and increases phosphorylation of AQP2 at Ser-256 and Ser-269. Metformin activates adenosine-monophosphate-activated protein kinase (AMPK) to phosphorylate AQP2. Clopidogrel inhibits the P2Y_12_ receptor (P2Y_12_-R) and increases adenylyl cyclase activity, resulting in AQP2 upregulation. Fluconazole upregulates AQP2 by increasing phosphorylation and abundance and by inhibiting RhoA. Statins inhibit RhoA, resulting in increased AQP2 expression in the apical membrane.

Other agents shown in Table 2 can exert antidiuretic effects in animal models of NDI, but no cases of hyponatremia associated with their use have been reported (Liamis et al., 2008). Generally, these drugs upregulate AQP2 expression in the collecting duct without modulating V2R expression (Figure 2).

The phosphodiesterase-5 inhibitor sildenafil citrate reduced polyuria and increased AQP2 expression in rats with lithium-induced NDI (Sanches et al., 2012). Sildenafil citrate may increase nitric oxide and prevent degradation of cGMP, resulting in increased AQP2 abundance in the apical membrane (Bouley et al., 2005). However, no reduction of urine volume or increase in urine osmolality was observed in a small number of NDI patients participating in clinical trials with sildenafil citrate (Moeller et al., 2013).

Metformin is a first-line antidiabetic agent and activates adenosine-monophosphate-activated protein kinase (AMPK). Klein et al. found that AMPK was expressed in the rat inner medulla and that metformin increased osmotic water permeability in association with AQP2 phosphorylation and trafficking to the apical plasma membrane (Klein et al., 2016). They also reported that in tamoxifen-induced V2R-knockout mice, urine concentration was improved by metformin treatment (Efe et al., 2016). However, hyponatremia is not reported to be associated with metformin despite widespread use in clinical practice.

Statins are β-hydroxy β-methylglutaryl-CoA (HMG-CoA) reductase inhibitors that are commonly used to reduce serum cholesterol concentrations. Li et al. showed that in AVP-deficient Brattleboro rats, simvastatin reduced polyuria in association with enhanced AQP2 trafficking through downregulation of Rho GTPase activity (Li et al., 2011). AQP2 upregulation that bypassed the AVP-V2R signaling pathway was also induced by other statins including lovastatin, rosuvastatin, cerivastatin, and fluvastatin (Procino et al., 2011). However, a recent epidemiologic study disproved the association between statins and hyponatremia (Skov et al., 2021).

Like EP3, P2Y_12_ receptor (P2Y_12_-R) signaling is mediated through Gi in the collecting duct and can be inhibited by clopidogrel (Zhang et al., 2015b). Zhang et al. reported that in rats with lithium-induced NDI, clopidogrel attenuated polyuria as a result of increasing adenylyl cyclase activity and AQP2 protein expression (Zhang et al., 2015a). Although clopidogrel is frequently prescribed for atherosclerosis prevention, its use has not been associated with hyponatremia.

Erlotinib inhibits the tyrosine kinase activity of the epidermal growth factor receptor (EGFR) and is used for treatment of non-small-cell lung cancer and pancreatic cancer (Gridelli et al., 2010). Cheung et al. reported that in mice with lithium-induced NDI, erlotinib reduced polyuria and enhanced apical membrane expression of AQP2 by increasing phosphorylation of AQP2 at S256 and S269 and reducing phosphorylation of AQP2 at S261 (Cheung et al., 2016). However, erlotinib alone does not appear to induce hyponatremia in cancer patients.

Fluconazole, an azole antifungal agent, was shown to enhance urine concentration in mice. Its antidiuretic action was associated with AQP2 upregulation from increased AQP2 phosphorylation and abundance, as well as RhoA inhibition (Vukićević et al., 2019). However, fluconazole alone does not appear to induce hyponatremia in infectious patients.

## Conclusion

**Figure 3:**
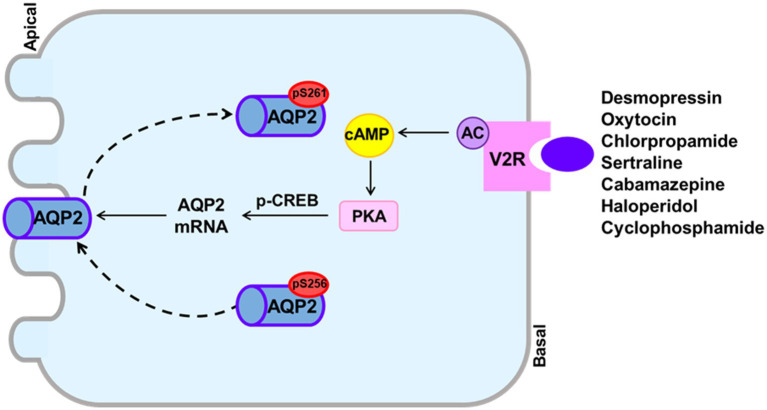
The canonical pathway from the vasopressin V2 receptor (V2R) to aquaporin-2 (AQP2) upregulation to induce drug-induced hyponatremia. Drugs that can cause hyponatremia bind to the V2R at basolateral membranes of collecting ducts and stimulate adenylyl cyclase activity, resulting in increased cAMP production. The cAMP/PKA signaling also induces enhanced AQP2 targeting to the apical membrane and increased AQP2 transcription probably through CREB phosphorylation.

Drug-induced hyponatremia is caused by renal water retention and was previously explained as induction of the SIADH. However, the SIAD is now considered to be the correct expression for the mechanism of drug-induced hyponatremia because the SIADH and NSIAD share the same clinical features, but present with different plasma AVP levels. Our literature review showed that SIADH underlies the hyponatremia induced by desmopressin, oxytocin, vincristine and ifosfamide. On the other hand, direct action of the drug on the kidney for AQP2 upregulation or induction of the NSIAD may underlie most cases of drug-induced hyponatremia. Previous *in vitro* and *in vivo* studies have shown that chlorpropamide, once thought to cause the SIADH, can cause hyponatremia by inducing the V2R-mediated pathway in the absence of AVP. We have shown that haloperidol, sertraline, carbamazepine, and cyclophosphamide act directly on the V2R in the collecting duct and upregulate AQP2 by inducing cAMP production (Figure 3). This finding contrasts with the mechanism of other experimental antidiuretic agents, which upregulate AQP2 by inducing various V2R-independent signaling pathways, and are not associated with hyponatremia. We believe that the canonical pathway from V2R to AQP2 upregulation has a pivotal role in drug-induced hyponatremia.

## Author Contributions

G-HK and SK designed the study and drafted and revised the paper. SK and CJ carried out the experiments. G-HK, SK, and CJ collected and analyzed the data. SK made the figures. All authors contributed to the article and approved the submitted version.

## Funding

This work was supported by a grant from the National Research Foundation of Korea (NRF-2020R1I1A1A01069274) to SK.

## Conflict of Interest

The authors declare that the research was conducted in the absence of any commercial or financial relationships that could be construed as a potential conflict of interest.

## Publisher’s Note

All claims expressed in this article are solely those of the authors and do not necessarily represent those of their affiliated organizations, or those of the publisher, the editors and the reviewers. Any product that may be evaluated in this article, or claim that may be made by its manufacturer, is not guaranteed or endorsed by the publisher.
